# Development of a model of three-dimensional imaging for the preoperative planning of TaTME

**DOI:** 10.1007/s10151-017-1724-7

**Published:** 2017-11-29

**Authors:** K. Sahnan, G. Pellino, S. O. Adegbola, P. J. Tozer, P. Chandrasinghe, D. Miskovic, R. Hompes, J. Warusavitarne, P. F. C. Lung

**Affiliations:** 1 Department of Colorectal Surgery, St Mark’s Hospital and Academic Institute, Imperial College University of London, Watford Road, Harrow, Middlesex HA13UJ UK; 2 Department of Surgery and Cancer, Imperial College, St Mary’s Hospital, London, UK; 3 Department of Medical, Surgical, Neurological, Metabolic and Ageing Sciences, University of Campania “Luigi Vanvitelli”, Naples, Italy; 4 Department of Surgery, University of Kelaniya, Kelaniya, Sri Lanka; 5 Department of Colorectal Surgery, Oxford University Hospitals NHS Foundation Trust, Oxford, UK

## Introduction

Since total mesorectal excision (TME) was first described in the early 1930s and later popularised by Heald [1], efforts have been made to standardise the technique, following the correct embryological planes and using appropriate landmarks. Laparoscopic and robotically assisted approaches to the rectum have gained popularity during recent years, compelling colorectal surgeons to develop their skills and knowledge. Transanal TME (TaTME) is a new addition to the approaches in rectal surgery. Despite being associated with several benefits in selected patients, TaTME requires advanced technical skills and, more importantly, knowledge of the pelvic structures, planes and spaces as they are encountered moving cephalad from the perineum. Magnetic resonance imaging (MRI) is the gold standard for imaging of the pelvis and pelvic floor, but understanding of relevant anatomy when performing a new technique may be hampered by difficulty in interpretation of two-dimensional (2D) images when considering three-dimensional (3D) structures. We describe a new tool that could help understanding of TaTME planes and preoperative planning.

## Materials and methods

Two cases were used to demonstrate our technique. Both patients were scheduled for TaTME and had undergone a preoperative MRI.

Standard axial T2-weighted spectral attenuated inversion recovery (SPAIR) and sagittal T2-weighted MRI sequences were obtained, and digital imaging and communications in medicine (DICOM) images were imported into a validated open-source segmentation software [2]. A specialist consultant gastrointestinal radiologist manually segmented the following structures: sphincter complex, rectosigmoid colon, levator plate, pelvis, mesorectal fascia, bladder, ureters, urethra, seminal vesicles and prostate. Each mesh was imported into another open-source system, MeshLab V1.3.3.1 as stereolithography (STL) files for mesh smoothing to be applied. Individual labels were applied to each anatomical structure.

## Results

Segmentation of patient images took approximately 15 min per case. A further 10 min was required for smoothing and applying colour and transparency of the anatomical structures to emphasise surgically relevant anatomy.

**Fig. 1:**
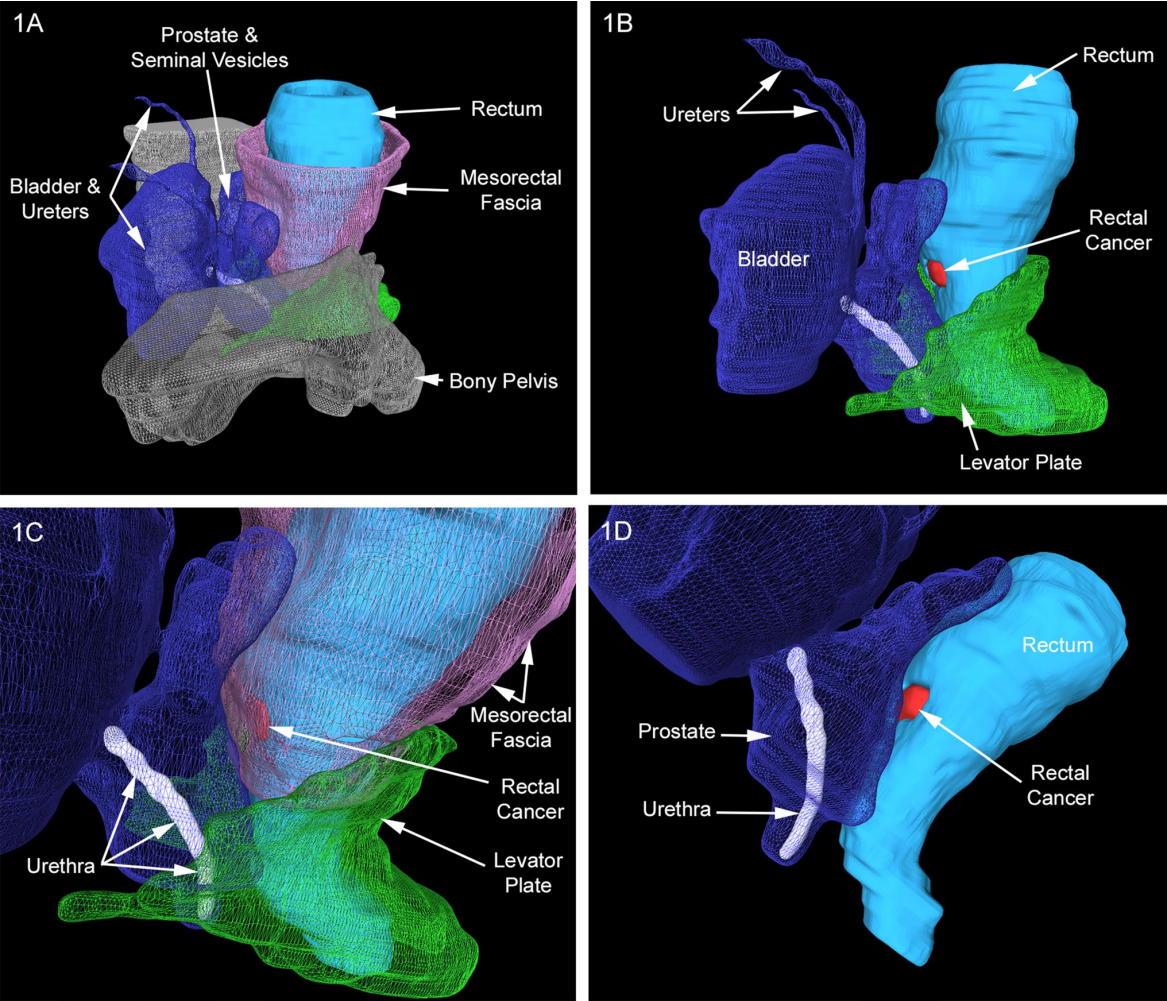
3D reconstructions for a patient with a low rectal cancer. **a** Overview of the pelvis and mesorectal fascia; **b** tumour penetrating the rectal wall; **c** tumour proximity to the prostate and adjacent urinary system; **d** angled view of tumour and the urethra

Patient 1 was a male with low rectal cancer who had TaTME. Relevant anatomy shown in Fig. 1a provides an overall overview of the pelvis and mesorectal fascia; Fig. 1b highlights the location at which the tumour penetrates the rectal wall; Fig. 1c demonstrates the proximity of the tumour to the prostate and adjacent urinary system, but also the clearance between them; and Fig. 1d is an angled view showing the relationship between the tumour and the urethra.

**Fig. 2:**
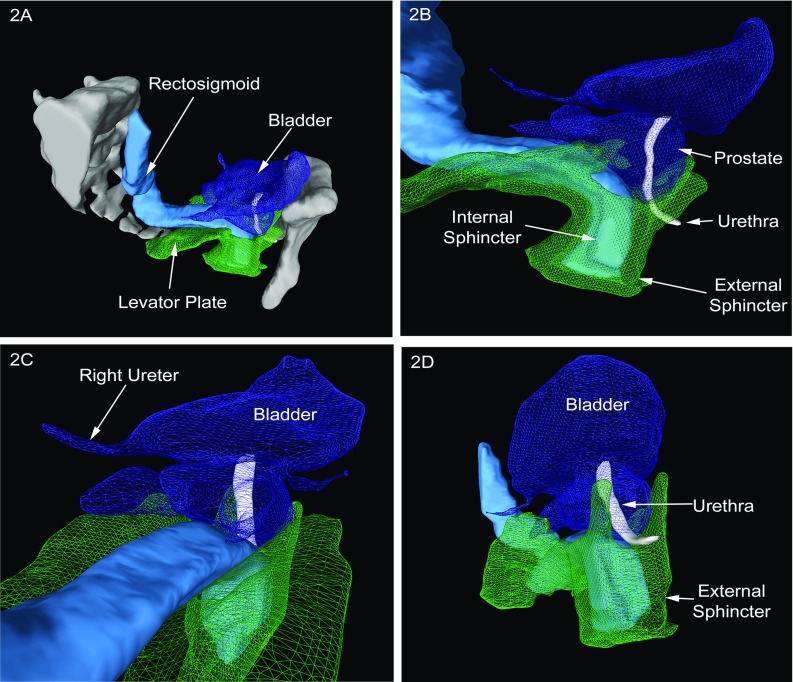
3D reconstructions for a patient with ulcerative colitis undergoing a completion proctectomy and ileoanal pouch formation. **a** Overview of the pelvis; **b** relation between internal sphincter/rectum and the prostate/urethra; **c** low rectum and the both ureters; **d** angled view of the sphincter complex and the urethra

Patient 2 was a male who had a combined single incision laparoscopy (SILS) and TaTME completion proctectomy and ileoanal pouch formation for ulcerative colitis. Figure 2a provides an overview of the anatomy showing a relatively straight and posterior direction of the rectum as it descends into the pelvis. Figure 2b provides insight into the relation between internal sphincter/rectum and the prostate/urethra. Distance between structures and relative proximity can be easily understood. Figure 2c shows the clearance between the low rectum and both ureters, whilst Fig. 2d shows an anterior oblique view of the sphincter complex and the urethra.

The 3D images can be rotated and the various structures inserted and removed so that the radiologist or surgeon can examine any structure form any angle, examine their relationships and determine distances and angles to facilitate safe dissection.

## Discussion

We provide two examples that demonstrate the utility of 3D modelling in surgical planning for TaTME, demonstrating how this technique is feasible and can be derived from manipulation of standard DICOM images from routine 2D MRI.

Transanal minimally invasive removal of the rectum, with or without TME, has gained popularity over the last decade. Specifically, TaTME offers better access to the distal, horizontal rectum in low-lying rectal cancers in patients with a narrow pelvis, bulky tumours or a large prostate, thereby allowing high-quality resection even under these circumstances [3]. Nevertheless, mastering the anatomy of the pelvis is demanding, and even surgeons familiar with TaTME may benefit from improved knowledge and understanding of important anatomical landmarks. Moreover, a TME might not always be necessary, for example in benign conditions such proctectomy for inflammatory bowel disease, further highlighting the importance of visualising the desired extent of resection ahead of surgery. TaTME involves a different approach to the routine for rectal surgery, necessitating a thorough appreciation of the pelvic anatomy to facilitate proficiency gains with the technique and minimise morbidity.

Improving the surgeon’s understanding of the relation of the pelvic organs to each other and of the pathology may protect patients from injury, especially at the start of the learning curve. An important risk is that of urethral injury, often in the pre-prostatic region, which may occur during the anterior dissection. The rate of injury was 1% in the international TaTME registry [4], but voluntary enrolment and selection bias may mean this is an underestimate of its true incidence. In particular, there are certain situations where the anatomy can be further distorted, such as post-chemoradiation [4], and in these instances adjunctive imaging through 3D reconstructions could be beneficial. Another potential cause for morbidity with TaTME is vaginal wall injury, which may also occur during anterior rectal dissection. Other smaller structures, such as the neurovascular bundle of Walsh, with capsular arterial branches, or the autonomic nerves, pose similar challenges in TaTME surgery. Both structures are difficult to appreciate using routine rectal cancer sequences, but where deemed necessary, dedicated sequences may be obtained to delineate this anatomy, allowing for 3D reconstruction. Dissection in a plane deep to the endopelvic fascia can result in injury to the inferior hypogastric plexus and bleeding from presacral veins. Surgeons must be familiar with the concept of the false “pneumodissection” plane and avoid following a plane deep to the nerve plexus [5].

Another matter to be considered carefully is the ideal route of specimen extraction. It is pivotal to select patients that may benefit from transabdominal extraction of the specimen, in order to avoid shearing of the mesentery with tumour cell exfoliation and shear stress to the marginal artery, with subsequent risk of ischaemia if an anastomosis is performed [4]. 3D allows one to follow each structure of the pelvis, including the urethra and surface of the prostate, detailing this delicate anatomy. The ability to rotate the 3D reconstruction into the same position as that of the patients on the table allows surgeons to assess the angles of dissection both anteriorly and posteriorly. This, combined with the facility to remove overlying structures, allows further appreciation of threatened margins, assessment of the optimum route of dissection and an awareness of abnormal anatomy.

Preoperative 3D modelling is a useful adjunct to routine preoperative planning. Notwithstanding the importance of adequate training and teaching in TaTME, it can also be a useful tool for the mentoring/proctoring surgeon to assess the knowledge of the mentee, and to discuss with them the detailed surgical strategy before the actual operation in each specific case. This is even more relevant when considering the possibility of accessing the reconstruction remotely. The measurement of anatomical factors such as the anorectal angle, anal canal length, buttock depth and interspinous distances would not only add further insight but allow the surgeon to assess the appropriate platform to be used. The 3D imaging can also be used to print patient-specific models, which could also be used during consultation with patients themselves, in order better to explain management strategies and obtain informed consent. Future models will aim to provide interactive elements so that the user can take full advantage of this platform, such as augmented or virtual reality, importantly, with haptic feedback. These innovations will revolutionise surgical rehearsal and also provide benefits during surgery itself, to improve training and patient outcomes.

3D modelling aids individualisation of treatment and surgical approaches. Identification of ideal surgical planes of excision, particularly in patients who do not need TME, in order to reduce the risk of collateral injury. It can be useful to address the extent of multivisceral resections in locally advanced cancers, and to assess patient suitability for the procedure. There are certain instances where conventional 2D MRI is favourable, such as in determining beyond TME approaches in cases where the circumferential resection margin is threatened. For example, one may identify particular parts of the mesorectal fascia or Denonvilliers’ fascia, which require en bloc excision, depending on tumour position. Future work will aim to improve segmentation techniques and add enhanced sequences to better understand this. In addition, comparison of anatomical factors such as tumour bulk, prostate volume, mesorectal volume and their influence on clinical outcomes would be interesting. Nevertheless, 3D rendering and the possibility of assessing each organ/structure separately represent an invaluable tool and adjunct.

## Conclusions

Surgeons currently use a combination of MRI scans, reports and discussion with radiologists to better understand anatomy and plan surgery. 3D reconstructions present an opportunity to improve a surgeon’s understanding of the information from 2D MRI, allowing for preoperative rehearsal of complex cases and to improve skill acquisition in innovative and existing surgical techniques. More experience using this technique is required before conclusions can be drawn on the impact of 3D imaging and its suggested benefits on technical error and complication.
